# Compassionate care during the COVID-19 pandemic

**DOI:** 10.1186/s12912-024-01827-x

**Published:** 2024-03-14

**Authors:** Jing Jing Su, Jonathan Bayuo, Rose S.Y. Lin, Arkers Kwan Ching Wong, Hammoda Abu-Odah, Qijun He, Ladislav Batalik

**Affiliations:** 1 School of Nursing, The Hong Kong Polytechnic University, Kowloon, Hong Kong SAR China; 2 Elaine Hubbard Center for Nursing Research on Aging, School of Nursing, University of Rochester Medical Center, Rochester, NY USA; 3 Department of Rehabilitation, University Hospital Brno, Brno, Czech Republic; 4 Department of Physiotherapy and Rehabilitation, Faculty of Medicine, Masaryk University, Brno, Czech Republic; 5 School of Journalism and Communication, Shanghai University, Shanghai, China

**Keywords:** Compassion, Compassionate care, Healthcare professionals, COVID-19, Pandemic, Qualitative

## Abstract

**Background:**

There was a substantial documented call for healthcare professionals to provide compassionate care during the COVID-19 pandemic and significant criticism voiced when it was lacking. This study aimed to explore perspectives on compassionate care among healthcare professionals providing care during the COVID-19 pandemic. The study focuses on healthcare professionals who participated in a wide range of COVID-19 measures, including testing, quarantine, diagnosis, and care provision (patients with COVID-19 or patients with other illnesses and comorbid with COVID-19).

**Methods:**

A qualitative design with an interpretative phenomenological analysis approach was used. Twenty frontline healthcare professionals (15 nurses and five physicians) who had worked in COVID-19 facilities in China were interviewed individually.

**Results:**

Participants stated that a commitment to ‘offering oneself’ and ‘balancing the advantages/disadvantages’ in providing care during the pandemic were key to alleviate population-level suffering. On a personal level, they described a desire for obtaining ‘mutual support’ and improving ‘professional competencies’ to safeguard their physical and mental well-being. Two professional competencies were notable: coping with grief and implementing infection control across the organization. Additionally, they emphasized the importance of receiving support from the health care organization, the public, and leaders in creating an ‘environment conducive to fostering compassionate care.’

**Conclusion:**

Healthcare professionals recognized the centrality of compassionate care during the pandemic which entailed a commitment to offering themselves, the balancing of advantages and disadvantages in order to find the best solution, as well as the need to safeguard themselves using professional competencies. Such findings can enrich the contemporary understanding of compassion, including when it is lacking. Support from the healthcare organization, the public, and leadership were crucial in fostering compassionate care in healthcare professionals during the pandemic and in moving the field forward in the future.

**Supplementary Information:**

The online version contains supplementary material available at 10.1186/s12912-024-01827-x.

## Introduction

Compassionate care (CC) is a core ethical principle of care among healthcare professionals (HCPs) in providing quality care [1], which is highlighted in codes of ethics [2], practice guidelines [3], and health policies [3, 4]. It refers to an inner feeling of another person’s suffering and the action of helping [5, 6]. Evidence suggests providing CC not only benefits patients but also has reciprocal benefits for HCPs. Compassionate care is associated with improved patient compliance, enhanced therapeutic relationships, increased satisfaction with care, and positive patient outcomes [1, 6, 7]. In providing CC, HCPs also report feeling accepted and appreciated by patients and colleagues, expressing reduced stress level, and having a higher sense of social safeness, and experiencing professional growth [8, 9].

Despite the recognized importance of CC, there are inherent difficulties in describing, exploring, or investigating this concept. Previous studies have explored HCP’s perspectives on CC among newly qualified nurses [10], nurses from palliative care settings [11], oncologists [12]. These studies agreed the importance and definition of CC, as well as acknowledged the challenges in mandating CC as the expression of CC by HCPs and the perception by patients can vary, and multiple factors could interfere with its delivery [6]. Providing CC during the pandemic presented additional complexities. HCPs were expected to risk their own health and wellbeing to deliver efficient, effective, and compassionate care amidst tremendous workplace pressure and pandemic-related restrictive measures [13]. While the general public was advised to avoid contact, HCPs were expected to directly engage with patients at risk of or infected with the virus. They had to address patient needs despite limited resources and emotional toll of witnessing high patient acuity and death rates [14]. Prolonged exposure to such stressful and threatening work environments may have impaired HCPs’ capacities to provide CC [15], which may explain why patients increasingly reported receiving care that lacked compassion and considered it as a crisis standard of care [16, 17]. 

When exploring CC during the pandemic, it is important to understand that individuals perceive CC differently, and even the same individual might perceive CC differently according to the occasion/context [18]. In normal circumstances, in-person therapeutic encounter allows the flow of compassion both within and between individuals [1], and CC is expressed by HCPs through genuine concern, therapeutic communication, wholehearted encouragement, and a warm presence that involves high levels of interpersonal interaction [19, 20]. However, during the pandemic, both HCPs and patients may have experienced frequent emotional reactions such as fear and uncertainty, exacerbated by a substantial decrease in human-human interaction, due to distancing and protective measures used in hospitals. Foreseeably, patients may have expressed appreciation for the sacrifice and efforts of HCPs and perceived a low level of interaction quality, communication, and perceived CC [18].

Limited empirical study has explored the experiences of frontline HCPs in providing CC during the pandemic. One qualitative study explored the influence of the pandemic (e.g., working while wearing PPE) on providing CC. Results revealed that COVID-19 has strongly and negatively affected their ability to deliver CC, resulting in moral injury and psychological difficulties [21]. Indeed, individuals who have experienced prolonged exposure to threatening and stressful working environments may have impaired CC competencies [22]. However, focusing on the effects of the pandemic on CC may yield findings that are negative in nature. It is increasingly recognized that compassion is emotionally protective and unlikely to cause burnout, and an array of factors/contexts may interfere with the capacity to express compassion [6]. To some extent, the pandemic was ripe for CC because suffering is a precondition for compassion to unfold [6].

In China, the perspectives on CC among healthcare professionals during the pandemic remains unclear. Ethical guidelines that are responsible for providing ethical directions in the core value of healthcare practice (Guiding Opinions on Establishing a System for Evaluating the Medical Ethics of Healthcare Professionals) do not provide clear description of CC [23]. One study explored Chinese nursing students perspectives on CC before the pandemic, stating the importance of empathy, communicating compassion, addressing individual needs [5]. Given the lack of information on CC among Chinese HCP during the pandemic, this study aimed to explore the perspectives of providing CC among frontline HCPs during the COVID-19 pandemic, where suffering was prevalent. It provides an ideal environment to expand the understanding of CC, how it is challenged, and to generate practical recommendations for future pandemic preparation. The study interviewed HCPs who participated in a wide range of COVID-19 measures, including testing, quarantine, diagnosis and care provision (patients with COVID-19 or patients diagnosed with other illnesses and comorbid with COVID-19).

## Methods

### Design

A qualitative design was used involving interpretative phenomenological analysis (IPA) that embraces phenomenology, hermeneutics, and idiography [24]. The aspects of phenomenology, hermeneutics, and idiography at the core of IPA focus on offering insights into how persons in a given context make sense of a given situation/ lived experience. These central tenets were considered congruent with the current study as this study sought to illuminate the experiences of HCPs regarding compassionate care provision during the pandemic. The aim of IPA was to explore the HCPs’ views as insiders and recognizes that researchers can reveal the insiders’ world through a process of interpretative activity [25]. This method highlights the dynamic and interactive characteristics of the data analysis process with both researchers’ and participants’ interpretations of the phenomenon being considered. It allows researchers to explore how participants understand their lived experience, which is contextually and socially bound, through the researchers’ interpretations to acquire reflective meaning of the participants’ experiences [26].

### Participants

Clinical HCPs (physicians and nurses) who worked in COVID-19 facilities in the past six months, such as designated COVID-19 treatment hospitals, inpatient units admitting COVID-19 patients, COVID-19 testing and screening facilities, fever clinics for COVID-19 diagnosis, and vaccination centers in Wuhan, China, were eligible. The inclusion of HCPs from different COVID-19 facilities was due to the extensive staff deployment during the pandemic, where professionals from different departments were reassigned to support the COVID-19 frontline. This study recruited physicians and nurses who provided direct patient care in COVID-19 facilities. The rational for this selection was based on the understanding that compassion is embodied and imbedded within the interpersonal context, encompassing the process of noticing, feeling, and acting to ease the patient suffering [27]. To maximize sample variation, HCPs with different socio-demographic backgrounds were recruited. The exclusion criteria were: HCPs in non-clinical roles in the back office and worked in COVID-19 facilities for less than two weeks (to ensure sufficient experiences in delivering CC and receiving feedback, as the COVID-19 incubation period lasts for 14 days).

### Sampling, sample size, and recruitment

A combination of purposive sampling and snowball sampling methods was used. This was due to the difficulty of recruiting participants, especially HCPs, during the pandemic [28]. The researchers disseminated the study information through research recruitment posters, social media, word of mouth, and peer referrals. Sampling adequacy was determined by the data saturation principle that two interviews were conducted after no new findings emerge in the interviews, attempting to confirm saturation [29].

### Ethics considerations

Ethics approval was granted by the ethics committees of Shanghai University (ECSHU2022046). Participants were told that they had the right to withdraw without any prejudice. Their confidentiality was protected in that no real name or name of the institution was collected, and data were accessible to the researcher for research purposes only. Written informed consent was obtained.

### Data collection

The data collection was conducted from May 2022 to December 2022. In-person and video-call interviews (e.g., Zoom) were used according to participants’ preferences. An appointment was made for HCPs who agreed to participate, and interviews were conducted in a comfortable and private setting with minimal interruption. This allowed the conversation to happen in a natural setting, for example, in participants’ homes or quiet gardens, within the context where the phenomenon occurs as part of the phenomenon itself (Babchuk, 2019). During the interviews, the principal investigator (female nurse with a doctoral degree in nursing) and another researcher followed the interview guide in asking open-ended and probing questions (see Table 1). The research questions were generated based on researchers’ previous research experiences in CC and a literature review regarding professionals’ experiences during the pandemic. Each interview was audio-recorded and lasted between 30 and 60 min. Field notes were documented during and immediately after the interview.

### Data analysis

The IPA was used. The researchers conducted verbatim translation and checked it against the recordings for accuracy. The researchers familiarized themselves with the transcripts by reading, rereading, and contemplation to immerse in the data, understand the interview holistically and identify meaningful words, phrases, or sentences. Nvivo was used for data management. The researchers made initial codes for the content and interpretations of participants’ transcripts. Data were coded line-by-line where margin notations with labels were made, and the referenced text was highlighted. Coding was performed as a cyclical process. The initial codes were modified and refined over the analysis period. The researcher analyzed the relationships across the codes and gathered the same ideas into sub-themes and themes. The sub-themes and themes were cross-checked with the transcripts for consistency. Consensus over coding and theme generation was attained through ongoing discussion among the researchers.

### Trustworthiness

The rigor of this study was ensured by following the five rigor principles of IPA [30]. Balanced integration was ensured by continually comparing the themes against the transcripts to ensure the interpretations were grounded in participants’ narration. Openness was improved by ensuring transparency, that researchers reviewed the analysis and interpretation until reaching a consensus. Concreteness was obtained by supporting the research findings with verbatim transcripts to position readers in the participants’ context. Resonance and actualization were improved by relating the study findings to implications on the research and clinical practice.

**Table 1:** Interview guide

1. How would you describe/define compassionate care (CC) during the pandemic?
2. Describe a situation, when you feel you or other HCPs have carried out CC when caring for a patient during the pandemic.
3. Describe a situation, if there has been one, when you feel CC was missing during the pandemic.
4. What are the barriers to providing CC during the pandemic? 5. What are the facilitators for providing CC during the pandemic?
6. Any suggestions for improving CC among HCPs?

## Results

Twenty frontline HCPs participated in this study, including fifteen nurses and five physicians. In terms of their roles during COVID-19, six participated in polymerase chain reaction (PCR) testing for COVID-19, four worked in hospital units admitting COVID-19 patients, three worked in designated COVID-19 hospitals, three worked in fever clinics for COVID-19 diagnosis, three worked in a COVID-19 intensive care unit (ICU), and one worked in a community COVID-19 quarantine center. There was a lack of participants from vaccination centers during study recruitment since most Chinese (88%) had received two doses of vaccine by March 2022 [31]. The average age was 31.58 (SD = 4.1) years; a majority (65%) were female. One participant had diploma education, ten had bachelor’s degrees, eight had master’s degrees, and one had a doctoral degree, with an average length of work experience of seven years. The detailed participants information is presented in Appendix 1.

The themes from the interviews were ‘offering oneself’ which stands at the core of CC, and was embodied by ‘obtaining mutual support,’ ‘balancing CC and containment measures,’ ‘improving professional competencies,’ and ‘creating an environment conducive to CC.’ More illustrative quotes are presented in Table 2.

### Offering oneself

#### Offering oneself: obligation and dedication

Most participants described experiences of CC as ‘voluntarily offering themselves’ to alleviate the suffering (e.g., infection and death) associated with COVID-19. They delineated CC during the pandemic as a shared commitment and responsibility among health professionals to live-out the principle of ‘if not me, then who’. Participants described the challenges associated with offering themselves in the context of rapidly increasing infected patient loads, healthcare system crisis, facing the unknown or even death.

I kept observing statistics and reports of COVID-19. It was very bad. I felt I should go to the frontline, instead of staying at home to write articles. After I went to the frontline, I felt more at ease. (COVID-19 ICU, nurse)

During the pandemic, I can accept extra workload that is beyond my role when my colleagues and leaders need me. (PCR testing, nurse)

#### Self-sacrifice for the greater good

According to participants, they tried to offer themselves to patients beyond a specific cohort and attempted to extend themselves to the population, as a whole. Thus, some participants regarded offering themselves during the pandemic as self-sacrifice for the benefit of not only their patient, but the community or the country—the greater good. Some participants linked this gesture of offering oneself to potential/indirect benefits for their loved ones who might be spared from the infection. Some participants stated that they felt the pandemic fostered a self-effacing, collective, and altruistic mentality in the healthcare system and the world more broadly.

It’s a shared commitment. Both I and my wife (a nurse) joined the frontline. Sacrifice small families to protect the big community. (PCR testing, physician)

I think when facing the pandemic, you see hope and the good side of humanity. People bravely share responsibilities to fight the pandemic in a selfish modern world. (Fever clinic for diagnosis, nurse)

Nonetheless, some participants shared situations where they or their colleagues withdrew themselves from CC provision due to their heavy workload or a need for self-protection. At the same time, participants were unwilling to harshly criticize this self-withdrawn attitude/behavior because they also expressed an understanding that work stress during the pandemic could prohibit CC. Several participants mentioned avoiding CPR as an example.

I think I did not do well in compassion. When we just started to admit COVID patients, it was new to us, and I generally did not want to be so close to them. They did not wear masks and were on ventilators. This seemed quite contradictory. (COVID-19 hospital, nurse)

Talking about a lack of CC, it comes to me when encountering dying patients who require CPR. We would normally perform it, but during the pandemic, we did not (to avoid transmission). (COVID-19 ICU, nurse)

### Mutual understanding

Participants elaborated on the importance of mutual understanding in providing CC during the pandemic: standing in other’s shoes (e.g., colleagues, patients, general public) despite the self-risking nature of care provision, constant containment policy changes, extreme working environments (e.g., fieldwork wearing PPE), and vast care demands in providing CC. They expressed that they were willing to risk their own wellbeing in order to enhance the wellbeing of others. And they also wished for and appreciated others who were involved in the pandemic (e.g., leaders, colleagues, patients, public) and could reciprocate with understanding to foster CC. They shared various scenarios among HCPs or between professionals and patients where they provided CC through the expression of mutual understanding and support. Many participants appreciated the mutual understanding among colleagues such as understanding the difficulty in cooperating with sudden staff deployment, negative emotions (uncertainty, anxiety, fear), unfamiliar work environment, new infection control workflow, and resource restraints. Such mutual understanding was considered crucial in equipping themselves with the courage and professional skills to provide CC.

I talked to my peers whenever I got frightened to figure out how to set the room: clean area and semi-contaminated area; how to prepare myself when crossing those areas. (COVID-19 hospital, nurse)

When fully equipped with PPE and N92 mask, it’s hard to breathe. I often had colleagues who started vomiting after working for a while and had to hold it until another colleague took over the shift (COVID-19 inpatient unit, nurse).

They also appreciated receiving mutual understanding and support from their patients when CC was provided. Participants elaborated that the heavy workload due to the pandemic required them to integrate infection control standards into every procedure, including but not limited to, ward management with multi-zones (clean area, observation area, quarantine area), surgery procedure and PCR testing. Participants sincerely wished that patients would show understanding by allowing professionals to explain procedures, cooperate with them and trust them. They also shared some incidences where patient reciprocated compassion through expressions of gratitude, helping staff by cleaning their room, complimenting the CC behaviors on social media, and verbal affirmations.

We all know pandemic containment is not easy, when everyone should use personal protection, avoid crowds, and follow surveillance protocol. Mutual understanding, please. It’s hard work for us. We understand patients, and they should understand us. (COVID-19 ICU, nurse)

Our ICU unit was redesigned to admit severe COVID-19 patients. No visitors/caregivers allowed, which means patients could not access food and daily life necessities. So we HCPs shared our meals and necessities with them. Later, when they became mobile; they helped us like cleaning. (Nurse)

When mutuality of understanding was lacking, participants shared complex and contradictory feelings that they would feel bad about patients, doubt/blame themselves, and at the same time defend themselves.

I have been working in this quarantine center for two months, every day staying in my room alone. I should have changed to another position by one month. I understand the staffing shortage, but it was not good for my mental health. In the worst moments, I would wonder if you have forgotten about me and the fact that there is still someone here. (Quarantine center, physician)

### Balancing compassionate care and containment measures

Participants unanimously shared that reinforcing restrictive pandemic containment measures are crucial and should be carried out with compassion to weigh and balance the advantages and disadvantages for each individual. Following those measures was essential to save lives and reduce suffering and could be considered an expression of compassion. However, there is a need to consider the impact of these measures on the individual and compassionately implement them. Participants shared that pandemic protocols can lead to patient isolation, disruptions in lifestyle, and health problems (e.g. stress). Participants expressed that providing CC requires that it be done with thoughtful consideration to decrease risks to the patient’s health. For instance, participants shared how pandemic practices such as needing to be fully contained in PPE and barring visitors exacerbated patients’ sense of social isolation. This issue was amplified in psychiatric units, where participants shared that patients often rejected them when they wore PPE or became frustrated when family visitors were prohibited. Participants also shared that patients were angry and non-cooperative with the containment policies, such as mandatory quarantine, mandatory PCR tests, travel restrictions, and having no visitors. Participants emphasized the need to be compassionate in implementing policies to prevent conflict.

I wear PPE, and then patients would be scared and say do not touch me. One (patient with schizophrenia) rejected assessment and treatment from us very vigorously. Our last resort is to use physical restraint. It hurts their dignity but we have to provide assessment and treatment and wear PPE. (Psychiatric unit admitting COVID-19 patients, nurse).

Rules are dead, people should be flexible. We reinforced no-visitor rules, and family members were worried. We would create short videos of patients for their families to comfort them. (COVID-19 hospital, nurse)

Participants expressed that a patient’s health status, education level and socioeconomic background could influence their perception of and appreciation for CC. Professionals could tailor their approach to that patient’s care to provide the best care.

Be thoughtful to the risk population, like older adults or pregnant women. Address their concerns adequately. (PCR testing, physician)

They also shared situations where professionals were harsh in reinforcing the pandemic containment measures and they regarded such incidences as examples of non-compassionate.

An advanced-stage cancer patient was admitted to our emergency department. She was wearing an oxygen mask. A nurse performed a throat swab to test for COVID-19 and the patient was having difficulty breathing, her oxygen saturation kept dropping and the nurse continued with the nasal swab. I really do not understand the point of insisting on a nasal swab. (Emergency unit admitting COVID-19 patients, nurse)

### Professional competencies in pandemic coping

Participants emphasized the professional competencies that enabled them to personally cope with workplace stress during the pandemic and to deliver CC. Two competencies were highlighted by participants to help them safeguard themselves mentally and physically in providing CC: coping with grief and implementation of infection control protocols throughout the organization.

You have to show your professionalism. You have to provide CC and explain your care actions. You have to show how carefully and diligently you followed the procedures. Then they would believe the necessity of your work. (Fever clinic, nurse).

You have to be professionally competent and confident. You are leading the team to provide care. Then you won’t be panicking or defeated easily. (COVID ICU, physician)

#### Coping with grief

Participants stated the importance of coping with grief in CC by recalling sad memories where care was limited by the complexity of the pandemic and subsequent adverse events or even patient death. They also shared disheartening events where colleagues died due to COVID-19.

Suddenly we are so close to death, sometimes patients or family members just die. Words appeared to be meaningless. (Nurse, inpatient unit admitting COVID-19 patients)

A professor-level physician in our hospital, had been entrusted with more medical responsibility and resources, suddenly died due to COVID. This was so disheartening. His today can be your tomorrow. When you wear your uniform, you put everything behind you (Nurse, fever clinic for diagnosis).

We discharged a patient who was hospitalized here twice. Later, he couldn’t access healthcare services. During the pandemic, he died. We heard the news and we remembered him; everyone was low in spirit. (PCR testing, nurse)

#### Implementing infection control across the organization

The participants emphasized the importance of implementing infection control measures across the organization. They suggested standardizing the physical environment, such as setting up negative pressure rooms, and clinical workflow/protocols to reduce suffering and death. By implementing consistent and effective infection control measures, professionals can minimize the risk of transmission and ensure that patients receive CC promptly.

The workflow should be standardized, when we identified positive cases, we need to make sure no spread within the center and ensure timely referral. (Quarantine center, physician)

It’s disheartening when people ignore the regulations. Hospitalized patients are high-risk populations, and no visitors were allowed. But some family members forcefully walked in. (Inpatient unit for COVID-19 patients, nurse)

The majority of the patients in our department got infected, and we needed a negative pressure room and professional PPE. (Nurse, psychiatric inpatient unit admitting COVID-19 patients)

### Creating an environment conducive to compassionate care

Participants acknowledged the importance of a working environment conducive to flourishing CC, including a focus on the concept at the leadership and management level and recognition of activities that fostered CC. They appreciated the joint efforts of leaders and managers in combating COVID-19, especially when leaders joined/witnessed the frontline work and supported the staff.

Our hospital has prioritized staffing in our ICU department. In a severe staffing shortage, the head nurses were redeployed to our unit to support us. We had four head nurses who joined us and worked day and night shifts. (COVID-19 ICU, nurse)

They shared a sense of pride when they or their colleagues received recognition from health/government officials or the general public for their dedication.

Our team received city-level awards for our dedication. We were strongly supported in resources and staffing by our community and hospital. (Nurse, COVID-19 hospital)

**Table 2:** Additional quotations to support the themes

Theme/subtheme	Quotes
Offering oneself *Self-sacrifice for the greater good*	How many times in a person’s life do you encounter a situation where our country needs you? I signed a waiver of assumption of risks and indemnity agreement to do this. (COVID-19 in-patient unit, nurse)
Mutual understanding	In our quarantine center, I tried to satisfy their physical and mental needs. And they would be more cooperative with the regulations. Not just telling them ‘you should……’. (Quarantine center, physician) The most touching thing is that the relationship is closer in our department. We are more like family now, after all, we have experienced life and death together. We promised to go in together, come back together alive, and we just need to be safe and sound. (COVID-19 inpatient unit, nurse)
Balancing CC and containment measures	In our ICU, resources were limited. If we were busy and patients wanted to talk, we would prioritize pharmacological treatment over chatting with them. We have to admit our limitations and focus on more crucial things. (COVID-19 ICU, physician) Physical violence may happen if we reinforce the policies aggressively (PCR testing, physician).
Creating an environment conducive to CC	We were not specialized in infectious diseases and were unprepared for the pandemic. Our leader kept telling us the steps and tips and everything became more and more standardized. (PCR testing, nurse)

## Discussion

Participants demonstrated a broad view in defining and implementing CC and acknowledged the centrality of CC as a core value that should be practiced, but also helped sustain them as HCPs throughout this challenging period. The findings of this study demonstrate that HCPs viewed CC as an essential value, which is composed of interconnected moral dimensions unified by a willingness to offer themselves to alleviate suffering. They equated CC with offering themselves, which was based on their obligation and dedication to the health of the population and being eager to use their professional capacity and ethos amidst a pandemic. In practice, this took the form of a desire to obtain mutual understanding, balance containment measures and CC in order to achieve the best outcome, enhance professional competencies, and the need for a conducive environment that allows HCPs compassion to flourish. This attitude of offering oneself echoes the core attributes of CC documented in the literature whereby awareness of a person’s suffering drives caring behaviors and attitudes [32]; but also, requires the dedication and self-sacrifice of the HCPs. The findings are consistent with the literature that reported HCP’s greater sense of CC during the pandemic and revealed that the system/conditions challenge them to provide CC [18].

Additionally, HCPs felt it was important that professionals be personally vulnerable to enhance CC in patient care, while also being cognizant of the limitations of the medical system and the need for mutual understanding (between colleagues, patients, general public, and leaders). They shared professional commitment to relieve suffering and saving lives by risking their health for the greater good, which called for mutual understanding. This affirms findings from other studies suggesting that compassion cannot be mandated, as it flows inwards and outwards, as well as reciprocally between individuals and the practice setting [33]. Prolonged workplace burnout may have impaired CC [5], which could explain the importance participants placed on mutual understanding. These findings extend previous research reporting on the importance of vulnerability on the part of patients and understanding on the part of the HCPs. Findings show that recognizing professionals’ vulnerability is also important, as is understanding towards HCPs on the part of the patient. Interestingly, while compassion is non-conditional, participants felt there were certain patient behaviors that enhanced their expression of compassion.

Another key finding from this study was the importance participants placed on balancing CC and containment measures, which requires wisdom, critical thinking, flexibility, and individualized care in following regulations. The literature emphasizes the importance of HCPs recognizing the uniqueness of each individual and responding with compassion and tailored care [34]. However, this differs from the literature’s emphasis on emotional investments of showing warmth, reflective listening, using patient’s dialect, and individualized teaching [5, 35], as the essentials during the pandemic became less emotionally invested. The key question centered on when to follow pandemic containment measures or follow the clinical best practice protocol, and how to combine both considerations and the challenges in conflicting situations. In other words, following pandemic protocols is CC, as well as bending the rules and not putting policy and procedure before the person. The findings differ from the literature that reported HCPs were providing crisis standards of care. Instead, the study revealed a shift from prioritizing patient satisfaction during regular times to focusing on patient survival during the pandemic. It may, to some extent, echo the literature relating CC to ‘tough love’ where professionals strategically help patients facing the harsh reality [36].

Further, the current findings demonstrated that HCPs included in this study strived to improve professional competencies to provide CC. Recognizing and responding to suffering with compassion are skills that are conducive to their professional development [37]. None of the participants in the study associated CC with emotional drainage, as excessive emotional resonance with patients’ suffering can lead to burnout [38]. Instead, they emphasized improving professional competencies to safeguard themselves physically and mentally to enable CC. The key aspects were coping with grief while witnessing deterioration and death and implementing infection control across the organization.

Healthcare professionals believe that leadership and managerial support play a crucial role in influencing the practice of CC [39]. It becomes more important for hospitals and clinics to cultivate a compassionate working culture to match with HCPs’ expressed competencies in providing CC. In doing so, professionals did not need to compromise their personal values of CC in keeping performance in-line with institutional requirements during the pandemic. In concordance with literature, the findings suggest that fostering CC is not only dependent on motivating individual professionals or training in compassion skills but, rather, creating an organizational compassionate culture [40, 41]. With the increasing complexity of staffing deployment and role/workload demands found in this study, this could impact the development and/or preservation of compassion among professionals. In other words, how professionals, in an unprecedented level of engagement, experienced compassion could largely determine how the healthcare system will face a pandemic in the future. Dedication and commitment from leaders, as well as recognition from the system and society, is the best reward for frontline professionals and crucial in shaping the attitude and ultimate CC behaviors of current and future professionals.

The findings from this study revealed frequent mentioning on sacrifice for the greater good and extensive discussion of prevalent suffering compared to a previous study conducted in China before pandemic. The previous study revealed strong focus on empathy, communication, individualized care needs, and role modelling from senior nurses [5]. This shift in description can be explained by the widespread suffering caused by the COVID-19 pandemic, which has inspired HCPs to risk their own wellbeing to meet the care demands of the healthcare system and public. Another noteworthy observation is that none of the participants mentioned the word “love” as a synonym for CC when describing their feelings and actions related to CC. This may be influenced by Chinese culture, which tends to be reserved in expressing emotions or explicitly saying ‘love’ [42], which contrast with other studies that considered love as a moral attribute of compassion [32].

### Implications

Healthcare professionals have recognized the importance of CC as an integral part of delivering quality care during a pandemic crisis by committing themselves to the greater good of population health. However, it is crucial to acknowledge that fostering CC during a pandemic goes beyond merely training healthcare professionals. The institutional values and work environment of healthcare systems can serve as either obstacles or facilitators in promoting CC. In addition to healthcare professionals, the public also plays a significant role in understanding and having appropriate expectations of CC from healthcare providers during a pandemic. It is important for the public to be aware that infection control measures, necessary to mitigate the spread of the virus, may impact the interpersonal aspects of compassion. This understanding can help manage expectations and ensure that the public recognizes the efforts and commitment of healthcare professionals, even when the usual expressions of compassion (e.g., therapeutic touch) are altered due to infection control measures. The findings of this study provide valuable insights into the provision of CC during the COVID-19 pandemic and can inform future pandemic responses. It is essential to conduct more empirical studies to confirm these findings and explore the similarities and differences in the delivery of CC across diverse social and cultural settings.

### Limitations

One limitation of this study is that HCPs were recruited from diverse institutions with different professional qualifications, roles, and obligations, and thus the findings may be too general to represent specific units (e.g., ICU unit). Despite achieving data saturation, this study only recruited five physicians whose views may be under-represented. Additionally, two factors may limit the generalizability of the findings to the overall situation of CC during COVID-19. Firstly; this study included only HCPs and the perspectives of patients are missing. Secondly; the sampling method may attract professionals who are more passionate about compassion. Another limitation that would be interesting to explore is how the differences in socio-demographics, such as gender dimension, healthcare settings, and professional backgrounds shape CC experiences. Methodologically, the phenomenological approach is to capture the experience in its entirety rather than a slice of it, because of which these aspects were under-addressed. More studies are need to allow more in-depth discussion regarding Asia/China context influence the operation of CC as current policies documents, ethics guidelines on CC are mostly generated in Western countries/healthcare settings.

## Conclusion

Healthcare professionals recognized the centrality of CC during the pandemic. They shared competence in compassion by expressing their commitment/ willingness to risk their own wellbeing for the wellbeing of others and balancing containment measures and CC in order to find the best solution for patients. They also expressed the need to safeguard themselves using professional competencies to enable CC. Such findings enrich contemporary understanding of compassion, including when it is lacking. Support from the healthcare system, the public and leadership is crucial in fostering compassion in HCPs during the pandemic and in improving pandemic management in the future.

### Electronic supplementary material

Below is the link to the electronic supplementary material.

Supplementary Material 1

## Data Availability

Data will be available upon reasonable request to the corresponding author.

## References

[1] Tehranineshat B, Rakhshan M, Torabizadeh C, Fararouei M (2019). Compassionate care in Healthcare System: a systematic review. J Natl Med Assoc.

[2] Sinclair S, McClement S, Raffin-Bouchal S, Hack TF, Hagen NA, McConnell S, Chochinov HM (2016). Compassion in Health Care: an empirical model. J Pain Symptom Manag.

[3] Compassion. in practice: nursing, midwifery, and care staff our vision and strategy, [https://www.england.nhs.uk/wp-content/uploads/2012/12/compassion-in-practice.pdf].

[4] Francis R. Report of the Mid Staffordshire NHS Foundation Trust public inquiry: executive summary. In., vol. 947; 2013.

[5] Su JJ, Masika GM, Paguio JT, Redding SR (2020). Defining compassionate nursing care. Nurs Ethics.

[6] Malenfant S, Jaggi P, Hayden KA, Sinclair S (2022). Compassion in healthcare: an updated scoping review of the literature. BMC Palliat Care.

[7] Zhou YM, Callejas MLA, Li YW, MacGeorge EL (2023). What does patient-centered communication look like? Linguistic markers of provider compassionate Care and Shared decision-making and their impacts on patient outcomes. Health Commun.

[8] View the code. of ethics for nurses [https://www.nursingworld.org/practice-policy/nursing-excellence/ethics/code-of-ethics-for-nurses/].

[9] Matos M, McEwan K, Kanovsky M, Halamova J, Steindl SR, Ferreira N, Linharelhos M, Rijo D, Asano K, Marquez MG (2022). Compassion protects Mental Health and Social Safeness during the COVID-19 pandemic across 21 countries. Mindfulness (N Y).

[10] Horsburgh D, Ross J (2013). Care and compassion: the experiences of newly qualified staff nurses. J Clin Nurs.

[11] Devik SA, Enmarker I, Hellzen O (2020). Nurses’ experiences of compassion when giving palliative care at home. Nurs Ethics.

[12] Cameron RA, Mazer BL, DeLuca JM, Mohile SG, Epstein RM (2015). In search of compassion: a new taxonomy of compassionate physician behaviours. Health Expect.

[13] Wiljer D, Charow R, Costin H, Sequeira L, Anderson M, Strudwick G, Tripp T, Crawford A (2019). Defining compassion in the digital health age: protocol for a scoping review. BMJ Open.

[14] Cartolovni A, Stolt M, Scott PA, Suhonen R (2021). Moral injury in healthcare professionals: a scoping review and discussion. Nurs Ethics.

[15] Sinclair S, Raffin-Bouchal S, Venturato L, Mijovic-Kondejewski J, Smith-MacDonald L (2017). Compassion fatigue: a meta-narrative review of the healthcare literature. Int J Nurs Stud.

[16] Hofmeyer A, Taylor R, Kennedy K. Fostering compassion and reducing burnout: how can health system leaders respond in the Covid-19 pandemic and beyond? Nurs Educ Today 2020, 94. 10.1016/j.nedt.2020.104502 PMC7295512 32980180

[17] Lake ET, Narva AM, Holland S, Smith JG, Cramer E, Rosenbaum KEF, French R, Clark RRS, Rogowski JA (2022). Hospital nurses’ moral distress and mental health during COVID-19. J Adv Nurs.

[18] De Rosis S, Gilmore KJ, Nuti S (2023). Reverse compassion: value-in-use and value-in-context of healthcare services during crisis. Tqm J.

[19] Renzi S, Fallanca F, Zangrillo A, Tresoldi M, Landoni G, Angelillo P, Pepe G, Pajoro U, Maestranzi G, Yacoub MR (2020). Caring with compassion during COVID-19. Palliat Support Care.

[20] Chu XR, Jaggi P, Louis JS, Sinclair S. Initial validation of a patient-reported Compassion measure in a Mandarin-speaking Long-Term Care Patient Population. J Nurs Meas 2023. 10.1891/JNM-2022-0097 37353318

[21] Egan H, Connabeer K, Keyte R, Tufte-Hewett A, Kauser S, Hussain M, Regan H, McGowan K, Mantzios M. I didn’t feel like I was a doctor’: a qualitative interview study exploring the experiences and representations of healthcare professionals’ capacity to deliver compassionate care and to practice self-care during the Covid-19 pandemic. Psychol Health 2023. 10.1080/08870446.2023.2174260 36760181

[22] Petrocchi N, Ottaviani C, Couyoumdjian A (2014). Dimensionality of self-compassion: translation and construct validation of the self-compassion scale in an Italian sample. J Ment Health.

[23] Guiding Opinions on Establishing a System for Evaluating the Medical Ethics of Healthcare Professionals. [http://www.nhc.gov.cn/jcj/s7692/200903/58597e2f0e5140d9b5767949de409e89.shtml].

[24] Smith JA (1996). Beyond the divide between cognition and discourse: using interpretative phenomenological analysis in health psychology. Psychol Health.

[25] Smith JA (2011). Evaluating the contribution of interpretative phenomenological analysis: a reply to the commentaries and further development of criteria. Health Psychol Rev.

[26] Smith JA, Fieldsend M. Interpretative phenomenological analysis. American Psychological Association; 2021.

[27] Khoury B (2019). Compassion: embodied and embedded. Mindfulness.

[28] Kobakhidze MN, Hui JI, Chui JI, Gonzalez A. Research disruptions, New Opportunities: re-imagining qualitative interview study during the COVID-19 pandemic. Int J Qual Meth 2021, 20.

[29] Hennink M, Kaiser BN (2022). Sample sizes for saturation in qualitative research: a systematic review of empirical tests. Soc Sci Med.

[30] de Witt L, Ploeg J (2006). Critical appraisal of rigour in interpretive phenomenological nursing research. J Adv Nurs.

[31] The proportion of. people fully vaccinated with the COVID-19 vaccine reaches 88.01% [https://www.gov.cn/xinwen/2022-03/26/content_5681691.htm].

[32] Blomberg K, Griffiths P, Wengstrom Y, May C, Bridges J (2016). Interventions for compassionate nursing care: a systematic review. Int J Nurs Stud.

[33] Chadwick R. Compassion: hard to define, impossible to mandate. Bmj-Brit Med J 2015, 351. 10.1136/bmj.h3991 26224485

[34] Su JJ, Paguio JT, Masika GM, Wang MA, Redding SR. Learning compassionate care: experiences of nursing students. Nurse Educ Pract 2021, 53. 10.1016/j.nepr.2021.103092 34049091

[35] Bramley L, Matiti M (2014). How does it really feel to be in my shoes? Patients’ experiences of compassion within nursing care and their perceptions of developing compassionate nurses. J Clin Nurs.

[36] Tierney S, Seers K, Tutton E, Reeve J (2017). Enabling the flow of compassionate care: a grounded theory study. Bmc Health Serv Res.

[37] Tracy SJ (2020). Let’s talk: conversation as a defining moment for the Communication Discipline. Health Commun.

[38] Lu H (2020). Exploring the role of Incidental and Integral Compassion and Anger in Health Communication about Pollution. Health Commun.

[39] Pavlova A, Paine SJ, Sinclair S, O’Callaghan A, Consedine NS (2023). Working in value-discrepant environments inhibits clinicians’ ability to provide compassion and reduces well-being: a cross-sectional study. J Intern Med.

[40] Sinclair S, Kondejewski J, Jaggi P, des Ordons ALR, Kassam A, Hayden KA, Harris D, Hack TF. What works for whom in compassion training programs offered to practicing healthcare providers: a realist review. Bmc Med Educ 2021, 21(1). 10.1186/s12909-021-02863-w PMC8403363 34454489

[41] Sinclair S, Kondejewski J, Raffin-Bouchal S, King-Shier KM, Singh P (2017). Can Self-Compassion Promote Healthcare Provider well-being and compassionate care to others? Results of a systematic review. Appl Psychol-Hlth We.

[42] Caldwell-Harris C, Kronrod A, Yang J (2013). Do more, say less: saying I love you in Chinese and American cultures. Intercult Pragmat.

