# Supplementary material for: Compassionate care during the COVID-19 pandemic
Source: BMC Nurs. 2024 Mar 14;23:173. doi: 10.1186/s12912-024-01827-x (PMC10938786; doi:10.1186/s12912-024-01827-x)
Supplement: Supplementary file 1 — Supplementary Material 1 [file 12912_2024_1827_MOESM1_ESM.docx]

Appendix 1. Characteristics of the Participants

|  | Age | Sex | Profession | Edu | COVID-19 facilities | Working experience (years) | Deployed from |
| --- | --- | --- | --- | --- | --- | --- | --- |
| 1 | 28 | Female | Nurse | Master | PCR testing | 5 | General practice |
| 2 | 30 | Female | Nurse | Bachelor | COVID-19 ICU | 7 | Gynecology Dept. |
| 3 | 29 | Male | Nurse | Master | PCR testing | 1 | Operating Theatre |
| 4 | 31 | Female | Nurse | Bachelor | COVID-19 inpatient | 8 | Respiratory Dept. |
| 5 | 30 | Male | Nurse | Master | PCR testing | 3 | Anesthesia Dept. |
| 6 | 29 | Female | Nurse | Master | Covid-19 inpatient | 3 | Geriatrics Dept. |
| 7 | 33 | Female | Nurse | Bachelor | PCR testing | 10 | Pharmacy Dept. |
| 8 | 36 | Male | Physician | Doctoral | designated COVID-19 hospitals | 8 | Vascular surgery Dept. |
| 9 | 33 | Female | Physician | Master | designated COVID-19 hospitals | 6 | Emergency Dept. |
| 10 | 37 | Male | Physician | Master | designated COVID-19 hospitals | 14 | Emergency ICU Dept. |
| 11 | 41 | Male | Physician | Bachelor | PCR testing | 17 | Ophthalmology Dept. |
| 12 | 31 | Female | Nurse | Master | Fever clinic | 10 | Oncology Depart. |
| 13 | 22 | Female | Nurse | Diploma | PCR testing | 1 | Rotate among Depts. as a junior nurse |
| 14 | 32 | Female | Nurse | Bachelor | COVID-19 ICU | 11 | ICU |
| 15 | 30 | Female | Nurse | Bachelor | Fever clinic | 6 | Medical Dept. |
| 16 | 30 | Female | Nurse | Bachelor | Fever clinic | 6 | Geriatrics Dept. |
| 17 | 37 | Male | Physician | Bachelor | Quarantine center | 12 | Community Health Center |
| 18 | 31 | Female | Nurse | Master | COVID-19 ICU | 6 | Hepatobiliary Surgery Dept. |
| 19 | 32 | Male | Nurse | Bachelor | COVID inpatient | 7 | Psychiatric Dept. |
| 20 | 30 | Female | Nurse | Bachelor | COVID-19 inpatient | 5 | Medical Dept. |

PCR: Polymerase chain reaction, ICU: intensive care unit,
